# First isolation and genotyping of *Toxoplasma gondii* strains from domestic animals in Tunisia

**DOI:** 10.1038/s41598-021-88751-1

**Published:** 2021-04-29

**Authors:** Arwa Lachkhem, Lokman Galal, Ibtissem Lahmar, Karine Passebosc, Homayoun Riahi, Nicolas Plault, Marie-Laure Dardé, Aurélien Mercier, Hamouda Babba

**Affiliations:** 1 Laboratoire de Parasitologie–Mycologie Médicale et Moléculaire (Code LR12ES08), Département de Biologie Clinique B, Faculté de Pharmacie de Monastir, Université de Monastir, Monastir, Tunisia; 2 INSERM, Université Limoges, CHU Limoges, IRD, U1094 Neuroépidémiologie Tropicale, Institut d’Epidémiologie et de Neurologie Tropicale, GEIST, Limoges, France; 3 Centre National de Référence (CNR) Toxoplasmose/Toxoplasma Biological Center (BRC), Centre Hospitalier-Universitaire Dupuytren, Limoges, France; 4 Centre de Maternité et de Néonatologie de Monastir, Monastir, Tunisia

**Keywords:** Biological techniques, Genetics

## Abstract

The isolation and molecular typing of *Toxoplasma gondii* strains provide an essential basis for a better understanding of the parasite’s genetic diversity, determinants of its geographical distribution and associated risks to human health. In this study, we isolated and genetically characterized *T. gondii* strains from domestic animals in Southern and coastal area of Tunisia. Blood, hearts and/or brains were collected from 766 domestic animals (630 sheep and 136 free-range chickens). Strain isolation from these samples was performed using mouse bioassay and genotyping was carried out with a multiplex PCR technique using 15 microsatellite markers. Thirty viable strains of *T. gondii* were successfully isolated from tissues of sheep (19/142) and chickens (11/33). In addition, 3 strains could be successfully genotyped from animal tissues for which mouse bioassay was unsuccessful. A large predominance of type II strains (n = 29) was found in the sampled regions, followed by type III (n = 3) and, for the first time in Tunisia, a single isolate of Africa 4 lineage from a sheep. Analyses of population genetics showed the presence of a divergent population of type II lineage in Tunisia, supporting limited recent migrations of strains between Tunisia and other countries of the world.

## Introduction

*Toxoplasma gondii* is a zoonotic apicomplexan parasite infecting humans and other warm-blooded animals including livestock^1,2^ and poultry^3,4^. The latter serve as an intermediate host, while felines are the final hosts of *T. gondii*^5^. It is estimated that *T. gondii* infects one third of the world's human population^6^. Humans are infected by ingesting tissue cysts from undercooked or raw meat or by accidental ingestion of oocysts released into the environment in feline feces^7,8^.

Toxoplasmosis is usually asymptomatic in healthy individuals. However, it can be serious or even fatal in the immunocompromised person^9–11^ or after congenital transmission^12,13^. It also has a significant economic impact, leading to numerous abortions in small farmed ruminants^14^.

First genotyping studies have suggested the existence of clonal populations for *Toxoplasma* with three main lineages: types I, II and III and rare recombinants strains^15^. In recent years, greater genetic variability has been demonstrated using multilocus markers. Thus, a large number of studies using these genotyping techniques have increased our knowledge of the distribution and global diversity of *T. gondii*^16–19^ with the identification of varying strain populations in different regions of the world. The worldwide distribution of *T. gondii* genotypes is well-known in Europe^20,21^, but remains little explored in Asia and Africa^22–24^. In Africa beside the intercontinental lineages type II and type III, African lineages named Africa 1, 2, 3 and recently Africa 4 have been identified using microsatellite markers (MS) or PCR-restriction fragment length polymorphism (RFLP)^4,24–28^. Other lineages or strain genotypes were rarely isolated, and the diversity of strains in the wild is still unknown^24^.

In Tunisia, previous studies mention a high prevalence of *T. gondii* in humans and domestic animals, but the genetic diversity of strains circulating in the country is still poorly known. One study performed on sheep meat from Tunis area, Northern Tunisia, showed the existence of types I, II and III and recombinant II/III strains^29^. Two genotyping studies were carried out on congenital cases of toxoplasmosis in the same region: the first study is a case of death associated with a recombinant genotype I/III^30^. In the second study, the genotypic analysis of the isolates from 14 cases of congenital toxoplasmosis by multilocus RFLP revealed the presence of different genotypes including type I, admixed genotypes with type I and type III alleles (I/III) or type I and type II alleles (I/II) and genotypes with multiallelic profiles for some markers (two to three alleles for the same locus)^31^.

However, the above-mentioned studies have been controversial as this genotypic diversity was contrasting with the one of other North African countries, in which type II was the predominant lineage and in which type I strains have never been isolated^24^. In addition, the uncommon proportions of mixed infections with abundance of type I alleles, raised concerns about a possible DNA contamination of samples with RH strain (a type I strain usually used in many laboratories as a PCR positive control), favored by the use of nested PCR techniques.

In a recent study, the strains involved in 4 cases of human congenital toxoplasmosis in Monastir area^32^ were isolated by mouse bioassay and genotyped using a highly discriminatory method based on the analysis of 15 microsatellite markers^16^. The four strains were found to be of type II lineage, a result more in tune with the diversity of strains expected to be found in a North African country^24^.

In view of these discordant results, it is therefore interesting to isolate strains from the domestic fauna in Tunisia and to genotype these strains using highly discriminating markers^16^, in order to determine which strains are truly circulating in this country. Notably, these markers have enough resolutive power to discriminate between different strains of the same lineage, enabling to distinguish type I laboratory strain (RH) from natural type I strains and therefore to detect DNA contamination issue.

In this context, the objective of this study was to describe the diversity of *T. gondii* strains circulating in Tunisia among domestic animals intended for human consumption. The sampling efforts were focused on two regions of the country: the coastal city of Monastir and the inland region of Gafsa. In order to estimate the extent of strain migrations between *T. gondii* populations from Tunisia and other regions of the world, we compared the genotypes of this study with those of previous studies, focusing mainly on other Mediterranean countries.

## Results

### Serological tests in domestic animals

The high sensitivity direct agglutination technique detected IgG antibodies against *T. gondii* in 175 (22.8%, 95% confidence interval CI 19.87–25.82) of 766 sera of animals. Seroprevalence was 22.5% [95% CI 19.24–25.76] and 24.3% [95% CI 17.09–31.51] in sheep and chickens, respectively.

### Isolated *T. gondii* strains

Viable strains of *T. gondii* were isolated from 13.4% (19/142) sheep and 33.3% (11/33) chickens. All the isolated strains were non virulent for mice.

Sheep isolates were designated by the BRC *Toxoplasma* code as TUN-Ovi ari-62 to 78 (with the exception of isolates Mo 184 and Mo 187, which were not retained at the BRC *Toxoplasma* for quality reasons and were therefore named by their field identifier), and in the same way chickens’ isolates were designated by TUN-Gal dom-34 to 42, 48 and 52 (Table S1). *Toxoplasma* PCR was positive in 100 animal tissue digests, 30 of which resulted in isolation of the strain after mice inoculation. Of the other 70 digests tested with qPCR, only 5 samples presented Cq values < 32 (from 25.4 to 31) which is the threshold to consider microsatellite genotyping.

### Microsatellite genotyping

Genotyping was performed on all live strains (30 strains) and on qPCR positive samples with Cq˂ 32 (5 DNA isolates for which the strain could not be genotyped on the mouse brains) (Table S2). Of 35 positive DNA extracts, 33 (21 sheep and 12 chickens) were successfully genotyped (94.3%). Thirty-two strains were fully genotyped by microsatellite analysis (15/15 MS markers) and one with 14/15 MS markers. Most of the *T. gondii* isolates (29/33; 87.9%) belonged to type II lineage. This allowed identifying in Monastir region, 13 type II isolates (10 sheep/3 chickens), three isolates of sheep type III, and, for the first time in Tunisia, an Africa 4 genotype in one ovine isolate. In the region of Gafsa, there were seven sheep isolates of type II and nine chicken isolates of type II of which two were single repeat variants at the W35 locus (allele 244 bp instead of 242 bp) of the ME49 type II reference strain (Table S2). Microsatellite analysis of isolates revealed no mixed infection in both studied regions.

**Figure 1:**
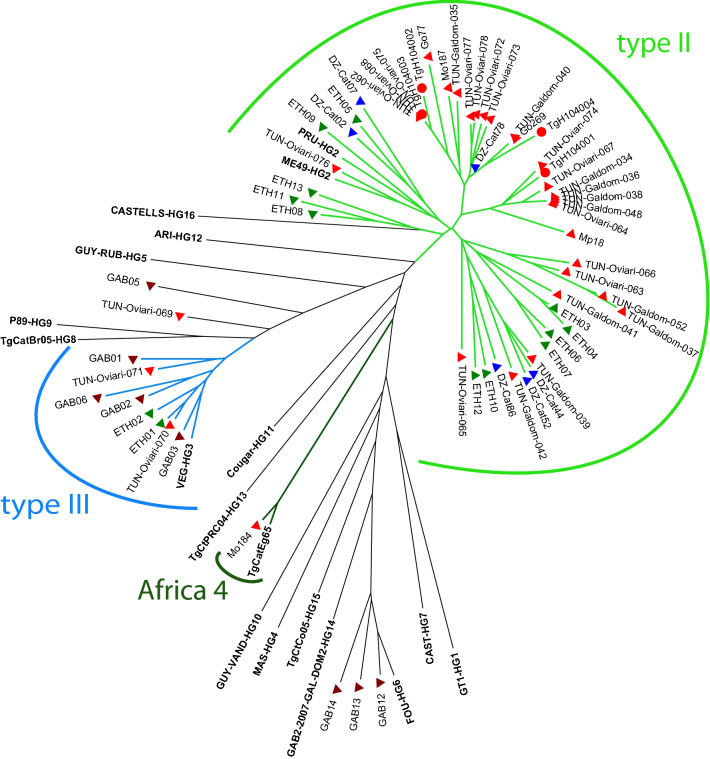
Neighbor-joining (NJ) tree of genotypes inferred from Cavalli–Sforza distances calculated from the data of 15 microsatellite markers for the 33 strains described in this study (red triangles) and a set of strains from previous studies: Human Tunisian strains (red circles), African strains (blue triangles for Algeria, green triangles for Ethiopia and brown triangles for Gabon) and reference strains representing the global diversity of *Toxoplasma gondii*. Reference strains and their respective haplogroups (HG) are indicated in bold letters when available (for details refer to Supplementary Table S2). Coloured branches in the NJ tree correspond to the *T. gondii* lineages found in Tunisia: type II lineage in light green, type III lineage in blue and Africa 4 lineage in dark green. This figure was drafted by L.G. in Adobe Illustrator CS6 (http://www.adobe.com/fr/products/illustrator.html).

In the NJ tree (Fig. 1), most Tunisian genotypes clustered into one of three groups corresponding to three clonal lineages: type II, type III and Africa 4. The type III strain TUN-Oviari-069 occupied an intermediate position between the type II and the type III clusters within the tree, likely due to an unusual allele at the M102 marker (176 instead of 190 ± one tandem repeat in most type III genotypes). Type II genotypes showed several subdivisions within the type II cluster in the NJ tree. Most type II genotypes from Tunisia (24/29) segregated from type II genotypes from other countries (Algeria, Ethiopia and USA) and gathered in three branches nearly exclusively composed of Tunisian genotypes. These branches included the Tunisian genotypes from human isolates^32^ from the same area. Other branches included type II genotypes from Tunisia (5/29) and other countries.

The paucity of strains belonging to type III and Africa 4 lineages in this study precluded performing extensive analysis of these lineages and hence the following analyses focused on strains of type II lineage.

**Figure 2:**
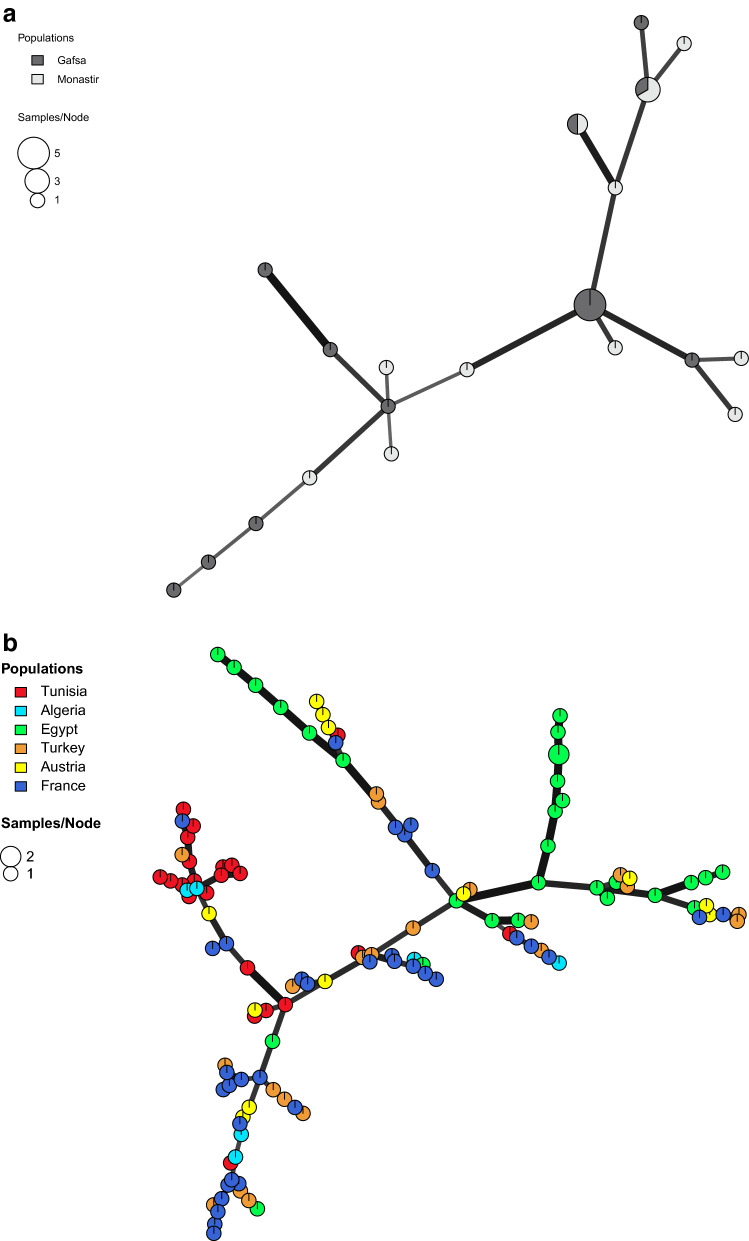
Minimum spanning network (MSN) representing the relationships between multilocus genotypes (MLG) of the type II lineage from (**a**) Monastir and Gafsa within Tunisia and (**b**) MLGs from Tunisia, Europe, Africa and Turkey. MSNs are based on MLGs defined by 15 microsatellite markers. The diameter of the point and the colour gave the number of strains and country of isolation respectively. Thick and dark lineages show MLGs that are more closely related to each other. The figures (**a**) and (**b**) were generated with the software RStudio 1.2.5042 (https://rstudio.com/products/rstudio/).

The MSN including type II genotypes from Monastir and Gafsa showed no geographical structure between the *T. gondii* populations from two Tunisian regions (Fig. 2a). Monastir and Gafsa had highly similar values of mean allelic richness, of 2.93 and 2.90, respectively.

The MSN including type II genotypes from Tunisia and 5 other countries (Algeria, Egypt, France, Turkey and Austria) showed that the majority of Tunisian genotypes cluster together and segregate from other geographical populations, with few exceptions (Fig. 2b). Notably, 2 of the 5 genotypes from Algeria included in this MSN clustered with the Tunisian group.

**Figure 3:**
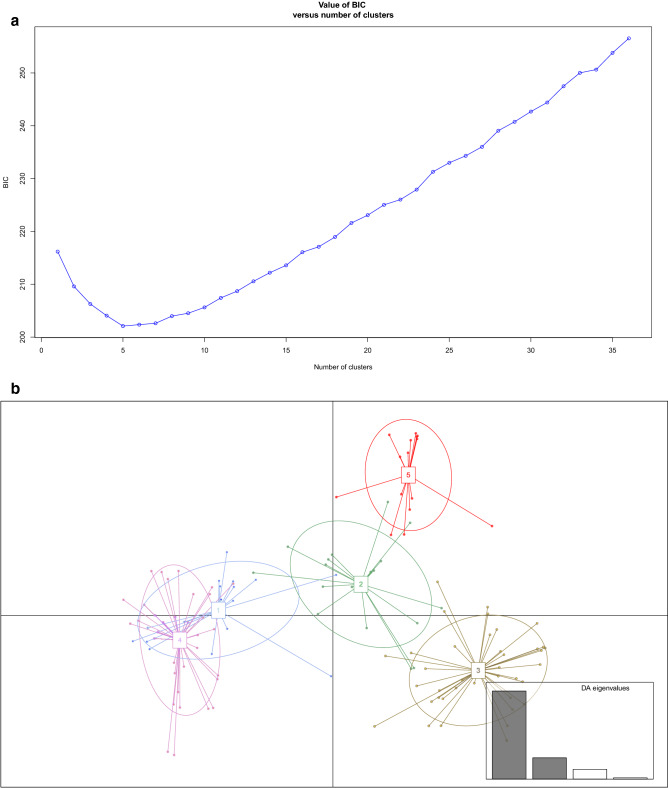

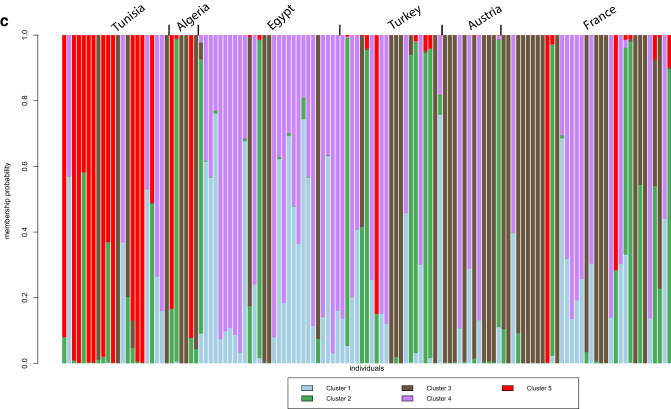
Genetic clustering of *Toxoplasma gondii* populations of type II lineage from Tunisia, Europe, Africa and Turkey utilizing the discriminant analysis of principal components (DAPC). (**a**) Bayesian information criterion (BIC) is provided for different numbers of clusters (from 1 to 35). (**b**) Scatterplot indicating axes 1 and 2 of the discriminant analysis of PCA-transformed data (DAPC). Individual clones are indicated by dots. Numbers and colours mention the five genetic clusters retained from Bayesian information criterion (BIC) values. (**c**) Barplots for the first two principal components of each individual estimated from DAPC. Individual strains are aligned along the x-axis and grouped according to the country of origin. Strains are assigned either to one cluster (each cluster is marked by a different colour) or to multiple clusters if their genotypes were admixed (indicated by multiple colours). The figures (**a**)–(**c**) were generated with the software RStudio 1.2.5042 (https://rstudio.com/products/rstudio/).

Using a selection model based on Bayesian information criterion (BIC) values, the optimal number of genetic clusters was K = 5 among type II genotypes from the six countries (Fig. 3a,b). The genetic cluster 5 was predominant among Tunisian genotypes (9/22) followed by cluster 3 (4/22), in addition to 9 admixed genotypes. Genotypes of Cluster 5 or with admixed profiles which cluster 5 were uncommon outside Tunisia and were found in Algeria (2/5), France (2/35) and Turkey (1/21) (Fig. 3c).

## Discussion

In this study, the main aim was to describe the genetic diversity of *T. gondii* strains among domestic animals intended for human consumption in Tunisia. Thirty strains were isolated and three DNA extracted directly from the tryptic digestion pellets for inoculation, which did not allow the isolation of strains. A total of 33 of these *T. gondii* strains and DNA isolates from free-ranging chickens and sheep were successfully genotyped (Table S2). Most of the *T. gondii* strains circulating in the study areas belonged to the type II clonal lineage (Fig. 1). For the region of Monastir, in addition to type II strains, three type III strains and, for the first time in Tunisia, an Africa 4 strain. This apparently higher diversity of lineages in Monastir compared to Gafsa can be attributed to the fact that the sheep slaughtered in this region often come from different governorates of the country. At the opposite, the region of Gafsa is a center for breeding livestock, so all slaughtered sheep are native to this region, which may explains the limited diversity of lineages observed in the samples from this region. However, these observations were made on a relatively small sampling, and should be confirmed by larger scale studies.

In addition, we found no difference in allelic richness between the two regions when considering type II lineage only, suggesting that the diversity of strains from Monastir is not obviously higher than that from Gafsa for this lineage.

The preponderance of the clonal type II lineage in this study is consistent with the results of previous studies that showed more generally the predominance of this lineage in North Africa^24^ and in other countries of the Mediterranean basin^33–36^. The dissemination of this lineage in these regions could be explained by the privileged trade exchange through both maritime and terrestrial routes for millennia, through the circulation of infected animals (cats, rodents and livestock herds)^23,24^.

The predominance of type II lineage is also in accordance with our data on human cases of congenital toxoplasmosis in Monastir area, which also revealed the presence of type II^32^. Type II genotypes were also found in poultry from Tunis, Northern Tunisia^37^.

The comparison of the type II genotypes from Monastir and Gafsa using a Minimum Spanning Network (Fig. 2a) showed no geographical structure between the *T. gondii* populations from two regions, a signal of recent migrations of this lineage within the country. Indeed, the sheep breeding centers connect the inland regions to the coast and the North regions of Tunisia by commercial movements of sheep herds. This connection is more accentuated during the sacrifice feast (Aïd Al-Adha) when an infected sheep from one region could be slaughtered in another and its offal could be consumed by cats leading to a regional dissemination of this lineage^25^.

Population genetics analyses using MSN and DAPC (Figs. 2b, 3b,c) supported a geographical isolation of *T. gondii* populations of type II lineage, with limited migrations of strains between Tunisia and other countries of the Mediterranean basin.

However, the rare genetic proximities highlighted in our analyses (Fig. 3c) could correspond to geographical proximities (Algeria) or exchanges related to the colonial history of Tunisia from the Ottoman Empire (Turkey) to the French colonization. The divergence of this Tunisian population of type II lineage could also be explained by a foundation effect caused by an imported type II strain. However, larger datasets of type II genotypes from different countries will be needed to test this hypothesis. Especially, additional sampling efforts in countries neighboring Tunisia such as Lybia and Algeria will be needed to have more robust estimates of the inter-country circulation of *T. gondii* strains.

In our study, the three detected type III isolates belonged to different regions located in the center of Tunisia (Sidi Bouzid region) and on the coast (cities of Monastir and Sousse). According to previous studies, Type III was the second most widespread genotype in the world, and was found in most sampled African countries^24,38^. Despite having the eight typing alleles typical of type III lineage, TUN-Oviari-069 showed some degree of divergence from the type III cluster in the tree (Fig. 1). This strain had an unusual allele at the M102 marker. M102 is a fingerprint marker exhibiting some degree of polymorphism compared to the 8 typing markers. However, this marker shows very little polymorphism in type III strains, as all type III strains genotyped to date have a fragment length of 190 ± one tandem repeat^4,17,25,34,39–41,50^. The fragment length of 176 found for this strain is much more commonly found in type II genotypes (including in Tunisia), and this genotype could therefore indicate a possible II/III recombinant strain. Whole genome analysis of this strain could be useful to test this hypothesis.

The detection of an Africa 4 strain isolated from a sheep for the first time in Tunisia confirms its spread across the African continent. Strains belonging to this lineage (Africa 4) have been repeatedly isolated from immune-suppressed African patients in France^24^ and more recently in poultry and rodents from Senegal and Benin, West Africa^4,38,42^. Other studies using RFLP markers showed the circulation of this lineage (RFLP genotypes of this lineage are known under the designation of ToxoDB # 20 and ToxoDB#137) in the Emirates, China, Sri Lanka as well as in East Africa^23,24,43^. Its geographic distribution follows an East–West axis connecting Asia and Africa.

In conclusion, this study revealed that the diversity of *T. gondii* strains in domestic animals from Tunisia is very similar to the diversity previously described in North Africa. Type II lineage was predominant, followed by type III and Africa 4. Population genetic analyses supported extensive circulation of strains within the country, but limited inter-country migrations of strains in the Mediterranean basin region in recent times.

However, further studies are needed on samples from domestic and wild animals collected in other unexplored regions of Tunisia and in neighbour countries for a more accurate description of the circulation of *T. gondii* strains in this region and the possible role of sheep herds in the spread of this parasite.

## Materials and methods

### Ethics statement

All animal experiments were performed according to the European Convention on the protection of animals used for scientific purposes (EU Directive 2010/63/EU) and approved by the local Experimental Animals Ethics Committee of the Faculty of Pharmacy, University of Monastir, Tunisia. Approval for the use of animals and all procedures was obtained from the Ethics Committee of the Faculty of Medicine, University of Monastir under ethics number IORG 0009738N°21/OMB 0990-0279. During the study, all methods were carried out in accordance with relevant guidelines and regulations and efforts were dedicated to minimize the suffering of used animals.

For butchers and chicken owners, the study was conducted in accordance with relevant guidelines and regulations and after ethical clearance obtained from the Ethics Committee of the Faculty of medicine, University of Monastir Tunisia (Code IORG 0009738N°21/OMB 0990-0279). Verbal informed consent was obtained and recorded from each participant after adequate explanation of the study purpose. Only butchers and chicken owners who agreed to participate were interviewed.

### Study area and sample collection

This study is an analytical cross-sectional study. It was conducted in two different areas in Tunisia: the first is located in Central-Eastern Tunisia in the coastal city of Monastir; while the second is located in the South-West of the country in Gafsa.

The study was carried out in compliance with the “Animal Research: Reporting of In Vivo Experiments” (ARRIVE) guidelines version 2.0. Thus, between September 2016 and May 2018, samples of blood, heart and/or brain were randomly collected from a total of 766 domestic animals reared in the region of Monastir and Gafsa. Only, animals aged of more than three months were included in this investigation.

A structured questionnaire which included the sex, age, species, breed, origin and living area of the animal, was used.

Blood and heart tissue samples were collected from 630 sheep (*Ovis aries*) slaughtered in slaughterhouses from two regions: Monastir and Gafsa (315 from each). Two breeds represented these animals: the Barbarine breed (n = 183) and the Queue fine de l’Ouest breed (n = 447).

In addition, samples of blood, brain and heart tissue were taken from each of the 136 free-range chickens (*Gallus gallus domesticus*) collected from farms and backyards (76 from Monastir and 60 from Gafsa).

Chicken blood was withdrawn from the wing vein using an insulin syringe (1 mL). These chickens were then marked with adhesive tape. The purchase of the animal and the removal of the heart and brain were done after a positive serology test. Sampling was conducted both in urban and rural localities in each region.

After centrifugation 10 min at 3000 rpm, sera were stored at − 20 °C until use. The hearts were removed sterilely, with a single-use scalpel blade, entirely for chickens and partly for sheep (apex of cardiac muscles) and the brains of the chickens were removed entirely by opening the cranial box. The tissue samples were placed in mixed saline solution of antibiotics with (1000 U/mL of penicillin and 10 mg/mL of streptomycin) and stored at + 4 °C before processing (Table S1).

### Serological examination

Sera were tested for *T. gondii* specific IgG antibodies in chicken and sheep using the highly sensitive direct agglutination test (DAT) (Toxo-Screen DA, BioMérieux, France) according to manufacturer instructions; sera was diluted to 1:40, 1:60, 1:180, 1:540, 1:1620 and 1:4000 with a seropositivity cut-off at 1:40 dilution titer.

### Bioassay of tissue samples for *T. gondii*

The isolation protocol was carried out as indicated previously with some modifications^25^. Brain and cardiac muscle tissues from seropositive domestic animals (≤ 50 g) were blended, homogenized in saline solution (0.9% NaCl) with a trypsin solution (0.25%) (Eurobio, Courtaboeuf, France) incubated in a shaker water bath at 37 °C for 90 min. The suspension was then filtered through two layers of gauze, the pellet was washed three times by centrifugation for 10 min at 2600 rpm, then resuspended in 0.9% NaCl, 200 µL of the suspension were stored at − 80 °C until DNA extraction. Finally, the digest pellets were incubated with antibiotics (1000 U/mL penicillin and 10 mg/mL streptomycin) at 4 °C overnight before inoculation.

About 1 mL of homogenate was inoculated intraperitoneally into each of four mice (female Swiss Webster mouse 20–25 g). Mice were monitored daily for clinical signs of toxoplasmosis.

Four weeks after inoculation, blood collection from mice was performed from the retro-orbital sinus of the eye for serological screening. The antibodies directed against *T. gondii* were determined by the DAT test (Toxo-Screen DA, BioMérieux, France).

Six weeks after inoculation, the brains of seropositive mice were removed aseptically. All brains and positive digests were transported to *T. gondii* Biological Resource Center (BRC *Toxoplasma*) of Limoges, for genotyping studies.

The brain was homogenized with 1 mL 0.9% NaCl using a 5 mL and 2 mL syringes with a 23 and 20 G needles respectively. After microscopic examination, 200 μL of each brain tissue were stored at − 20 °C until DNA extraction.

Strains were cryopreserved in liquid nitrogen with RPMI containing 10% FCS and 10% DMSO at BRC *Toxoplasma*, Limoges, France (http://www.toxocrb.com).

### DNA extraction and genotyping of *T. gondii* isolates

Genomic DNA was extracted from 200 μL of mouse brain tissue for each strains and directly from the digest of animal seropositive tissue, using the QIAamp DNA MiniKit (Qiagen, Courtaboeuf, France) according to the manufacturer's recommendations.

We firstly checked the presence of toxoplasmic DNA in the digest of seropositive animals by conventional PCR as mentioned in^32^.

Secondly, before genotyping we estimate the amount of *Toxoplasma* DNA from digests of PCR positive animal tissues for which we failed to isolate the strain using a real-time quantitative PCR (qPCR) targeting the 529 bp repeat region of *T. gondii* DNA fragment (GenBank accession number AF146527)^44^, as described previously^45^.

All brains from seropositive mice and animal tissue digests with a Cq value less than 32 by qPCR were subjected to genotyping analysis using 15 microsatellite markers, as previously described^16^. These markers included 8 typing markers (TUB2, W35, TgM-A, B18, B17, M33, IV.1, XI.1), showing little or no variation within lineages and 7 “fingerprinting” markers (M48, M102, N83, N82, AA, N61, N60) showing significant polymorphism variation within lineages.

### Genetic clustering

In order to quantify the extent of genetic distance between Tunisian populations and evaluate their position in relation to the previously described genetic diversity of *T. gondii* strains, a Neighbour-joining (NJ) tree was constructed from the genetic distances among individual isolates using Populations 1.2.32 (http://bioinformatics.org/populations/) based on Cavalli-Sforza and Edwards chord distance estimator^46^ and generated with MEGA 6.05 (http://www.megasoftware.net/history.php).

Reference strains representing the 16 T*. gondii* haplogroups (HGs) described to date^17,47^.

were used for comparison with strains from this study: GT1 (HG1), ME49 and PRU (HG2), VEG (HG3), MAS (HG4), RUB (HG5), FOU (HG6), CAST (HG7), TgCtBr5 (HG8), P89 (HG9), VAND (HG10), COUG (HG11), ARI (HG12), TgCtPRC04 (HG13), TgA105004 (HG14), TgCtCo5 (HG15) and CASTELLS (HG16). TgCatEg65^4,48^ was used as the reference strain for Africa 4 lineage. In addition, human strains previously isolated from cases of congenital toxoplasmosis in Tunisia^32^ were also included. Finally, a number of animal isolates from Algeria^49^, Ethiopia^50^, and Gabon^25^ were included in order to compare Tunisian strains to other African strains.

A Minimum spanning network (MSN) was generated using “Poppr” package^51^ (implemented in R environment) to evaluate the geographical segregation between the genotypes from Monastir and Gafsa. HP-Rare 1.1^52^ was used to compare allelic richness between the two regions using a rarefaction procedure.

A second MSN was generated in order to evaluate the extent of migrations of *T. gondii* strains between Tunisia and other regions of the world. The MSN included genotypes from this study and a set of previously published genotypes mainly originating from mediterranean countries (Algeria, Egypt, France, Turkey) and Austria.

Discriminant analysis of principal components (DAPC), implemented in the ADEGENET package in the R environment^53^, was performed to infer population subdivision within the same set of genotypes from Tunisia and other countries. This nonparametric approach (free from Hardy–Weinberg assumptions) makes no assumptions regarding data structure or underlying population genetics model, and is therefore suitable for organisms which display high levels of clonality such as *T. gondii*. In this model, genetic data is initially transformed using a principal components analysis (PCA), followed by a discriminant analysis (DA) to identify clusters. The optimal number of clusters (populations) is calculated using the *k*-means clustering algorithm, based on the Bayesian information criterion (BIC), which reaches its minimum when approaching the best supported assignment of individuals to the appropriate number of clusters. Individuals having less than 90% of probability of membership in a single cluster were considered as admixed^54^. R packages were run in R sofware version 3.4.0.

## Supplementary Information

Supplementary Tables.

## Data Availability

The datasets used and/or analyzed during the current study are available from the corresponding author on reasonable request.
