# Supplementary material for: First isolation and genotyping of Toxoplasma gondii strains from domestic animals in Tunisia
Source: Sci Rep. 2021 Apr 29;11:9328. doi: 10.1038/s41598-021-88751-1 (PMC8085010; doi:10.1038/s41598-021-88751-1)
Supplement: Supplementary file 1 — Supplementary Tables. [file 41598_2021_88751_MOESM1_ESM.docx]

| **Isolate**  **Supplementary Table S1: Epidemiological data, serology results of 33 *T. gondii* positive samples isolated from sheep and free-range chickens of Tunisia** | **BRC ^a^ code** | **Age**  **(Months)** | **Sex** | **Species** | **Region** | **Area of life** | **Samples** | | **DAT**  **(IU/mL)** |
| --- | --- | --- | --- | --- | --- | --- | --- | --- | --- |
|  |  |  |  |  |  |  | **tissue** | **Weight** |  |
| Mo21 | **TUN-Ovi ari-075** | >37 | M | *Ovis aries* | Monastir | Sidi Bouzid | heart | 10.5 g | 54 |
| Mo69 | **TUN-Ovi ari-069** | >37 | M | *Ovis aries* | Monastir | Monastir | heart | 50 g | 400 |
| Mo81 | **TUN-Ovi ari-071** | [3-12] | M | *Ovis aries* | Monastir | Sidi Bouzid | heart | 19.3 g | 400 |
| Mo117 | **TUN-Ovi ari-066** | >37 | M | *Ovis aries* | Monastir | Moknine | heart | 39 g | 54 |
| Mo133 | **TUN-Ovi ari-074** | [13-24] | M | *Ovis aries* | Monastir | Monastir | heart | 35 g | 400 |
| Mo156 | **TUN-Ovi ari-068** | [13-24] | M | *Ovis aries* | Monastir | Fahs | heart | 23.3 g | 54 |
| Mo180 | **TUN-Ovi ari-077** | >37 | F | *Ovis aries* | Monastir | Monastir | heart | 22 g | 400 |
| Mo184 | **-** | [3-12] | M | *Ovis aries* | Monastir | Sidi Bouzid | heart | 19.5 g | 400 |
| Mo187 | **-** | >37 | F | *Ovis aries* | Monastir | Sbeitla | heart | 18.6 g | 400 |
| Mo195 | **TUN-Ovi ari-078** | >37 | F | *Ovis aries* | Monastir | Sousse | heart | 26 g | 400 |
| Mo233 | **TUN-Ovi ari-070** | >37 | F | *Ovis aries* | Monastir | Sousse | heart | 17 g | 400 |
| Mo238 | **TUN-Ovi ari-073** | >37 | F | *Ovis aries* | Monastir | Sidi Bouzid | heart | 25 g | 400 |
| Mo258 | **TUN-Ovi ari-076** | [13-24] | M | *Ovis aries* | Monastir | Monastir | heart | 15.6 g | 400 |
| Mo260 | **TUN-Ovi ari-067** | [3-12] | M | *Ovis aries* | Monastir | Monastir | heart | 14 g | 400 |
| MP18 | **-** | <12 | F | *Gallus gallus domesticus* | Monastir | Monastir | heart and brain | 9 g | 162 |
| MP34 | **TUN-Gal dom-040** | <12 | M | *Gallus gallus domesticus* | Monastir | Ksiba | heart and brain | 13 g | 400 |
| MP56 | **TUN-Gal dom-039** | <12 | M | *Gallus gallus domesticus* | Monastir | Monastir | heart and brain | 17.5 g | 162 |
| Go15 | **TUN-Ovi ari-063** | [3-12] | M | *Ovis aries* | Gafsa | Gafsa | heart | 9.2 g | 400 |
| Go74 | **TUN-Ovi ari-062** | >37 | F | *Ovis aries* | Gafsa | Gafsa | heart | 11.3 g | 400 |
| Go75 | **TUN-Ovi ari-065** | >37 | F | *Ovis aries* | Gafsa | Gafsa | heart | 12.3 g | 400 |
| GO77 | **-** | >37 | F | *Ovis aries* | Gafsa | Gafsa | heart | 6.4 g | 400 |
| Go151 | **TUN-Ovi ari-064** | [13-24] | M | *Ovis aries* | Gafsa | Gafsa | heart | 31.6 g | 400 |
| Go269 | **-** | [3-12] | F | *Ovis aries* | Gafsa | Gafsa | heart | 12.2 g | 4 |
| Go304 | **TUN-Ovi ari-072** | >37 | F | *Ovis aries* | Gafsa | Gafsa | heart | 27 g | 54 |
| GP2 | **TUN-Gal dom-041** | >12 | M | *Gallus gallus domesticus* | Gafsa | Mdhila | heart and brain | 11 g | 400 |
| GP25 | **TUN-Gal dom-035** | >12 | F | *Gallus gallus domesticus* | Gafsa | Gafsa | heart and brain | 15 g | 400 |
| GP34 | **TUN-Gal dom-052** | <12 | F | *Gallus gallus domesticus* | Gafsa | Gafsa | heart and brain | 13.5 g | 400 |
| GP36 | **TUN-Gal dom-037** | <12 | F | *Gallus gallus domesticus* | Gafsa | Gafsa | heart and brain | 16.1 g | 400 |
| GP45 | **TUN-Gal dom-042** | <12 | M | *Gallus gallus domesticus* | Gafsa | Gafsa | heart and brain | 26 g | 162 |
| GP49 | **TUN-Gal dom-036** | >12 | M | *Gallus gallus domesticus* | Gafsa | Gafsa | heart and brain | 9.5 g | 400 |
| GP50 | **TUN-Gal dom-048** | >12 | M | *Gallus gallus domesticus* | Gafsa | Gafsa | heart and brain | 8.1 g | 400 |
| GP52 | **TUN-Gal dom-034** | >12 | F | *Gallus gallus domesticus* | Gafsa | Gafsa | heart and brain | 9.3 g | 400 |
| GP54 | **TUN-Gal dom-038** | <12 | F | *Gallus gallus domesticus* | Gafsa | Gafsa | heart and brain | 9.4 g | 400 |

M: Male; F: Female; g: gram; TUN: Tunisie; DAT: Direct Agglutination Test

^a^Toxoplasma Biological Resource Center; Strains not kept in BRC have no BRC code

| **Supplementary Table S2 : Real Time PCR and genotyping results of *T. gondii* DNA with 15 microsatellite markers from 33 isolates collected from sheep and free-range chickens of Tunisia** | | | | **Marqueurs microsatellites** | | | | | | | | | | | | | | |
| --- | --- | --- | --- | --- | --- | --- | --- | --- | --- | --- | --- | --- | --- | --- | --- | --- | --- | --- |
| **BRC ^a^ code or strain reference** | **Nature of genotyped samples for this study (Cq^b^)** | **Region of isolation** | **MS-Type ^c^/** **RFLP equivalent**  **genotype** | **TUB2** | **W35** | **TgM-A** | **B18** | **B17** | **M33** | **MIV.1** | **MXI.1** | **M48** | **M102** | **N60** | **N82** | **AA** | **N61** | **N83** |
| **GT1**  **(HG1)^d^**^(1)(2)^ |  | **USA** | **Type** I /  ToxoDB#10 | 291 | 248 | 209 | 160 | 342 | 169 | 274 | 358 | 209 | 168 | 145 | 119 | 265 | 087 | 306 |
| **Me49** **(HG2)**^(1)(2)^ |  | **USA** | **Type II** /  ToxoDB#1 | 289 | 242 | 207 | 158 | 336 | 169 | 274 | 356 | 215 | 174 | 142 | 111 | 265 | 091 | 310 |
| **PRU (HG2)**^(1)(2)^ |  | **France** | **Type II /** ToxoDB#3 | 289 | 242 | 207 | 158 | 336 | 169 | 274 | 356 | 209 | 176 | 142 | 117 | 265 | 123 | 310 |
| **VEG** **(HG3)**^(1)(2)^ |  | **USA** | **Type III** /  ToxoDB#2 | 289 | 242 | 205 | 160 | 336 | 165 | 278 | 356 | 213 | 188 | 153 | 111 | 267 | 089 | 312 |
| **MAS (HG4)**^(1)(2)^ |  | **France** | **Atypical** /  ToxoDB#17 | 291 | 242 | 205 | 162 | 362 | 169 | 272 | 358 | 221 | 166 | 142 | 111 | 332 | 095 | 338 |
| **GUY-RUB (HG5)**^(1)(2)^ |  | **French Guiana** | **Atypical** /  ToxoDB#98 | 289 | 242 | 205 | 170 | 360 | 167 | 274 | 356 | 223 | 190 | 142 | 109 | 259 | 085 | 312 |
| **FOU (HG6)**^(1)(2)^ |  | **France** | **Africa 1** /  ToxoDB#6 | 291 | 248 | 205 | 160 | 342 | 165 | 274 | 354 | 227 | 166 | 147 | 111 | 281 | 089 | 306 |
| **CAST (HG7)**^(1)(2)^ |  | **USA** | **Atypical** /  ToxoDB#28 | 291 | 242 | 205 | 158 | 342 | 167 | 276 | 356 | 211 | 168 | 147 | 119 | 279 | 087 | 306 |
| **TgCatBr05 (HG8)**^(1)(2)^ |  | **Brazil** | **Atypical** /  ToxoDB#42 | 291 | 242 | 205 | 160 | 362 | 165 | 278 | 356 | 237 | 174 | 140 | 111 | 265 | 089 | 314 |
| **P89 (HG9)**^(1)(2)^ |  | **USA** | **Atypical** /  ToxoDB#8 | 291 | 242 | 205 | 160 | 348 | 165 | 278 | 356 | 213 | 190 | 142 | 111 | 261 | 087 | 314 |
| **GUY-VAND (HG10)**^(1)(2)^ |  | **French Guiana** | **Atypical** /  ToxoDB#60 | 291 | 242 | 203 | 162 | 344 | 167 | 276 | 356 | 217 | 170 | 142 | 113 | 277 | 091 | 308 |
| **Cougar (HG11)**^(1)(2)^ |  | **Canada** | **Atypical** /  ToxoDB#66 | 289 | 242 | 205 | 158 | 336 | 169 | 274 | 354 | 219 | 174 | 151 | 119 | 259 | 079 | 332 |
| **ARI (HG12)**^(1)(2)^ |  | **USA** | **Atypical** /  ToxoDB#5 | 289 | 242 | 209 | 158 | 336 | 169 | 274 | 362 | 215 | 170 | 147 | 131 | 295 | 089 | 316 |
| **TgCtPRC04 (HG13)**^(1)(2)^ |  | **China** | **Chinese 1** /  ToxoDB#9 | 293 | 242 | 211 | 160 | 336 | 169 | 274 | 354 | 215 | 172 | 145 | 123 | 281 | 093 | 308 |
| **TgCatEg65**^(3)^ |  | **Egypt** | **Africa 4 /**  ToxoDB#20 | 291 | 242 | 203 | 156 | 336 | 165 | 274 | 354 | 223 | 174 | 130 | 109 | 303 | 099 | 310 |
| **TgA105004 (HG14)**^(1)(2)^ |  | **Gabon** | **Africa 3** /  ToxoDB#203 | 291 | 242 | 207 | 160 | 342 | 165 | 278 | 354 | 223 | 166 | 142 | 111 | 277 | 097 | 310 |
| **TgCtCo05 (HG15)**^(1)(2)^ |  | **Columbia** | **Atypical** /  ToxoDB#61 | 291 | 242 | 205 | 160 | 336 | 165 | 276 | 356 | 223 | 166 | 142 | 121 | 279 | 087 | 304 |
| **CASTELLS (HG16)**^(2)^ |  | **Uruguay** | **Atypical** /  ToxoDB#51 | 287 | 242 | 207 | 158 | 358 | 169 | 274 | 356 | 239 | 164 | 138 | 109 | 383 | 087 | 324 |
| **TUN-Ovi ari-062** | Mouse brain | Gafsa | **Type II (15/15)** | 289 | 242 | 207 | 158 | 336 | 169 | 274 | 356 | 215 | 184 | 140 | 109 | 275 | 103 | 312 |
| **TUN-Ovi ari-063** | Mouse brain | Gafsa | **Type II (15/15)** | 289 | 242 | 207 | 158 | 336 | 169 | 274 | 356 | 225 | 176 | 140 | 113 | 259 | 99 | 310 |
| **TUN-Ovi ari-064** | Mouse brain | Gafsa | **Type II (15/15)** | 289 | 242 | 207 | 158 | 336 | 169 | 274 | 356 | 215 | 182 | 140 | 109 | 285 | 105 | 310 |
| **TUN-Ovi ari-065** | Mouse brain | Gafsa | **Type II (15/15)** | 289 | 242 | 207 | 158 | 336 | 169 | 274 | 356 | 213 | 174 | 140 | 127 | 289 | 87 | 310 |
| **TUN-Ovi ari-066** | Mouse brain | Monastir | **Type II (15/15)** | 289 | 242 | 207 | 158 | 336 | 169 | 274 | 356 | 225 | 176 | 140 | 123 | 259 | 95 | 310 |
| **TUN-Ovi ari-067** | Mouse brain | Monastir | **Type II (15/15)** | 289 | 242 | 207 | 158 | 336 | 169 | 274 | 356 | 215 | 182 | 140 | 109 | 259 | 105 | 310 |
| **TUN-Ovi ari-068** | Mouse brain | Monastir | **Type II (15/15)** | 289 | 242 | 207 | 158 | 336 | 169 | 274 | 356 | 215 | 184 | 140 | 109 | 275 | 103 | 312 |
| **TUN-Ovi ari-069** | Mouse brain | Monastir | **Type III (15/15)** | 289 | 242 | 205 | 160 | 336 | 165 | 278 | 356 | 215 | 176 | 140 | 111 | 259 | 89 | 312 |
| **TUN-Ovi ari-070** | Mouse brain | Monastir | **Type III (15/15)** | 289 | 242 | 205 | 160 | 336 | 165 | 278 | 356 | 213 | 190 | 147 | 111 | 265 | 89 | 312 |
| **TUN-Ovi ari-071** | Mouse brain | Monastir | **Type III (15/15)** | 289 | 242 | 205 | 160 | 336 | 165 | 278 | 356 | 215 | 190 | 147 | 111 | 275 | 89 | 312 |
| **TUN-Ovi ari-072** | Mouse brain | Gafsa | **Type II (15/15)** | 289 | 242 | 207 | 158 | 336 | 169 | 274 | 356 | 215 | 184 | 140 | 109 | 285 | 107 | 310 |
| **TUN-Ovi ari-073** | Mouse brain | Monastir | **Type II (15/15)** | 289 | 242 | 207 | 158 | 336 | 169 | 274 | 356 | 215 | 184 | 140 | 109 | 287 | 115 | 310 |
| **TUN-Ovi ari-074** | Mouse brain | Monastir | **Type II (15/15)** | 289 | 242 | 207 | 158 | 336 | 169 | 274 | 356 | 215 | 182 | 140 | 109 | 279 | 111 | 310 |
| **TUN-Ovi ari-075** | Mouse brain | Monastir | **Type II (15/15)** | 289 | 242 | 207 | 158 | 336 | 169 | 274 | 356 | 215 | 184 | 140 | 109 | 275 | 103 | 312 |
| **TUN-Ovi ari-076** | Mouse brain | Monastir | **Type II (15/15)** | 289 | 242 | 207 | 158 | 336 | 169 | 274 | 356 | 221 | 174 | 142 | 109 | 265 | 101 | 310 |
| **TUN-Ovi ari-077** | Mouse brain | Monastir | **Type II (15/15)** | 289 | 242 | 207 | 158 | 336 | 169 | 274 | 356 | 215 | 184 | 140 | 109 | 291 | 103 | 310 |
| **TUN-Ovi ari-078** | Mouse brain | Monastir | **Type II (15/15)** | 289 | 242 | 207 | 158 | 336 | 169 | 274 | 356 | 215 | 184 | 140 | 109 | 291 | 99 | 310 |
| **Go77** | Tissue DNA extract (Cq 26.6) | Gafsa | **Type II (14/15)** | 289 | NA | 207 | 163 | 336 | 169 | 274 | 356 | 215 | 184 | 147 | 109 | 269 | 103 | 308 |
| **Go149** | Tissue DNA extract (Cq 29.3) | Gafsa | **ND (2/15)** | NA | NA | NA | NA | NA | NA | NA | NA | NA | 176 | NA | 109 | NA | NA | NA |
| **Mo184** | Mouse brain | Monastir | **Africa 4 (15/15)** | 291 | 242 | 203 | 156 | 336 | 165 | 274 | 354 | 223 | 174 | 130 | 109 | 297 | 99 | 310 |
| **Mo187** | Mouse brain | Monastir | **Type II (15/15)** | 289 | 242 | 207 | 158 | 336 | 169 | 274 | 356 | 235 | 184 | 140 | 109 | 281 | 107 | 312 |
| **Go269** | Tissue DNA extract (Cq 25.4) | Gafsa | **Type II (15/15)** | 289 | 242 | 207 | 158 | 336 | 169 | 274 | 356 | 215 | 184 | 140 | 109 | 295 | 105 | 310 |
| **TUN-Gal dom-034** | Mouse brain | Gafsa | **Type II (15/15)** | 289 | 242 | 207 | 158 | 336 | 169 | 274 | 356 | 215 | 182 | 140 | 109 | 285 | 105 | 310 |
| **TUN-Gal dom-035** | Mouse brain | Gafsa | **Type II (15/15)** | 289 | 242 | 207 | 158 | 336 | 169 | 274 | 356 | 219 | 184 | 140 | 109 | 287 | 103 | 312 |
| **TUN-Gal dom-036** | Mouse brain | Gafsa | **Type II (15/15)** | 289 | 242 | 207 | 158 | 336 | 169 | 274 | 356 | 215 | 182 | 140 | 109 | 285 | 105 | 310 |
| **TUN-Gal dom-037** | Mouse brain | Gafsa | **Type II (15/15), variant W35** | 289 | 244 | 207 | 158 | 336 | 169 | 274 | 356 | 213 | 176 | 142 | 113 | 259 | 99 | 310 |
| **TUN-Gal dom-038** | Mouse brain | Gafsa | **Type II (15/15)** | 289 | 242 | 207 | 158 | 336 | 169 | 274 | 356 | 215 | 182 | 140 | 109 | 285 | 105 | 310 |
| **TUN-Gal dom-039** | Mouse brain | Monastir | **Type II (15/15)** | 289 | 242 | 207 | 158 | 336 | 169 | 274 | 356 | 219 | 174 | 140 | 111 | 281 | 107 | 308 |
| **TUN-Gal dom-040** | Mouse brain | Monastir | **Type II (15/15)** | 289 | 242 | 207 | 158 | 336 | 169 | 274 | 356 | 215 | 184 | 140 | 109 | 295 | 105 | 310 |
| **TUN-Gal dom-041** | Mouse brain | Gafsa | **Type II (15/15)** | 289 | 242 | 207 | 158 | 336 | 169 | 274 | 356 | 213 | 176 | 140 | 125 | 267 | 103 | 310 |
| **TUN-Gal dom-042** | Mouse brain | Gafsa | **Type II (15/15)** | 289 | 242 | 207 | 158 | 336 | 169 | 274 | 356 | 233 | 174 | 140 | 111 | 287 | 91 | 314 |
| **TUN-Gal dom-048** | Mouse brain | Gafsa | **Type II (15/15)** | 289 | 242 | 207 | 158 | 336 | 169 | 274 | 356 | 215 | 182 | 140 | 109 | 285 | 105 | 310 |
| **TUN-Gal dom-052** | Mouse brain | Gafsa | **Type II (15/15), variant W35** | 289 | 244 | 207 | 158 | 336 | 169 | 274 | 356 | 213 | 176 | 140 | 113 | 259 | 99 | 310 |
| **GP13** | Tissue DNA extract (Cq 28.6) | Gafsa | **ND (4/15)** | NA | NA | 207 | NA | NA | 169 | NA | NA | 245 | NA | NA | NA | NA | 87 | NA |
| **MP18** | Tissue DNA extract (Cq 31) | Monastir | **Type II (15/15)** | 289 | 242 | 207 | 158 | 336 | 163 | 274 | 356 | 215 | 182 | 140 | 107 | 283 | 85 | 310 |

ND: genotype of strain not determined; NA: sequence not amplified; Ovi ari: *Ovis aries*; Gal dom: *Gallus gallus domesticus*

^a^ The strains (Mo 184, Mo 187) were kept at the laboratory of Parasitology, Monastir

**^b^** Cq : Quantification cycle

**^c^** MS-Type : Type defined by multilocus microsatellite genotyping

^d^HG: reference strains representing the 16 haplogroups of *T. gondii*

^(1)^Su, C. *et al.* Globally diverse Toxoplasma gondii isolates comprise six major clades originating from a small number of distinct ancestral lineages. *Proc. Natl. Acad. Sci.USA* **109**, 5844-5849 (2012).

^(2)^Lorenzi, H. *et al.* Local admixture of amplified and diversified secreted pathogenesis determinants shapes mosaic Toxoplasma gondii genomes. *Nat. Commun.* **7**, 10147, doi:10.1038/ncomms10147 (2016).

^(3)^Al-Kappany, Y. M. *et al.* High prevalence of toxoplasmosis in cats from Egypt: isolation of viable Toxoplasma gondii, tissue distribution, and isolate designation. *J. Parasitol.* **96**, 1115-1118, doi:10.1645/ge-2554.1 (2010).
